# Use of CentriMag for refractory cardiogenic shock in a puerperal woman: case report

**DOI:** 10.1590/1516-3180.2020.0775.18022021

**Published:** 2021-05-10

**Authors:** Paulo Manuel Pêgo-Fernandes, Augusto Scalabrini, Ludhmila Abrahão Hajjar, Priscila Berenice da Costa, Roberto Kalil, Fabio Biscegli Jatene

**Affiliations:** I MD, PhD. Full Professor, Thoracic Surgery Division, Instituto do Coracao, Hospital das Clinicas HCFMUSP, Faculdade de Medicina, Universidade de Sao Paulo, Sao Paulo, SP, BR; and Cardiothoracic Surgeon, Hospital Sírio Libanês (HSL), São Paulo (SP), Brazil.; II MD, PhD. Member of the Scientific Committee, Cardiology Center, Hospital Sírio Libanês (HSL), São Paulo (SP), Brazil; and Associate Professor, Emergency Clinic, Hospital das Clinicas HCFMUSP, Faculdade de Medicina, Universidade de Sao Paulo, Sao Paulo, SP, BR.; III MD, PhD. Medical Supervisor of Cardio-Oncology, Instituto do Coracao, Hospital das Clinicas HCFMUSP, Faculdade de Medicina, Universidade de Sao Paulo, Sao Paulo, SP, BR.; IV PhD. Registered Nurse, Thoracic Surgery Division, Instituto do Coracao, Hospital das Clinicas HCFMUSP, Faculdade de Medicina, Universidade de Sao Paulo, Sao Paulo, SP, BR.; V MD, PhD. Full Professor, Cardiology Division, Instituto do Coracao, Hospital das Clinicas HCFMUSP, Faculdade de Medicina, Universidade de Sao Paulo, Sao Paulo, SP, BR; and General Director of the Cardiology Center, Hospital Sírio Libanês (HSL), São Paulo (SP), Brazil.; VI MD, PhD. Full Professor, Cardiovascular Surgery Division, Instituto do Coracao, Hospital das Clinicas HCFMUSP, Faculdade de Medicina, Universidade de Sao Paulo, Sao Paulo, SP, BR; and Cardiovascular Surgeon, Hospital Sírio Libanês (HSL), São Paulo (SP), Brazil.

**Keywords:** Heart failure, Heart-assist devices, Extracorporeal membrane oxygenation, Myocarditis, CentriMag, ECMO, Puerperal, Refractory cardiogenic shock

## Abstract

**CONTEXT:**

Heart failure in Brazil is a major public health problem and, even with advances in treatment, it still presents high morbidity and mortality. As a treatment option, mechanical circulatory assist devices (MCADs) have greatly increased in importance over the last decade.

**CASE REPORT:**

This report concerns a case of refractory cardiogenic shock due to acute myocarditis in a 35-year-old puerperal female patient who presented with retrosternal pain, fatigue and dyspnea. At the hospital, she was diagnosed with myocarditis. There was no improvement in perfusion even after receiving dobutamine, intra-aortic balloon passage (IAB) and venoarterial extracorporeal membrane oxygenation (VA-ECMO). Therefore, it was decided to implant a MCAD (CentriMag). During hospitalization, recovery from the bi-ventricular dysfunction was achieved. The CentriMag device was removed 10 days after it had been implanted, and the patient was discharged after another 8 days. The myocarditis was proven to be due to the Coxsackie virus.

**CONCLUSIONS:**

The decision to implant a MCAD should be individualized, as patient profiles do not always match the indications in the guidelines and protocols. In this study, clinical discussion of the case among the medical and multi-professional teams was essential in order to be able to successfully reverse the patient’s severe clinical condition without sequelae, through using a CentriMag implant.

## INTRODUCTION

Worldwide, there were approximately 26 million people with heart failure in 2014. This therefore represents a major public health problem.^1^ In Brazil, the scenario is not different and, even with advances in treatment, heart failure still presents high morbidity and mortality. The incidence of heart failure in Brazil is 199 cases per 100,000 person-years, and the one-year mortality rate is 24.5% (95% confidence interval, CI, 19.4%-30.0%).^2^ Recent data from the Department of Information Technology of the Brazilian National Health System (DATASUS) have shown that in Brazil the number of heart failure deaths was 27,461 just in 2017.^3^

As a treatment option, mechanical circulatory assist devices (MCADs) have greatly increased in importance over the last decade. Although there are solid guidelines for indication of MCAD implantation,^4^ some particular cases still need to be studied, such as cases of refractory cardiogenic shock due to myocarditis. The objective of this case report was to present a case of refractory cardiogenic shock due to acute myocarditis in a young puerperal woman.

## CASE REPORT

This present case report was approved on April 13, 2020 (#1599; CAAE 30403220.7.0000.5461). A 35-year-old married female patient who was in the early puerperal period (childbirth in April 2019) sought emergency assistance with flu symptoms that she had had for approximately six days. At the emergency room, she was diagnosed with tonsillitis and was treated with azithromycin and prednisone. In the absence of symptom improvement, she presented retrosternal pain, fatigue and dyspnea. In a new medical evaluation, markers for myocardial necrosis were examined, with positive results, suggesting a diagnostic hypothesis of myopericarditis.

After admission to the coronary unit, she presented signs of low cardiac output with precordial pain, nausea and peripheral perfusion and was then referred to the advanced heart failure unit. She used vasoactive drugs (VAD), but without improvement in perfusion and clinical presentation. After her case has been discussed by the team, it was decided to perform intra-aortic balloon (IAB) passage. Because of progression of dysfunction and worsening of her general condition, it was decided to install peripheral venoarterial extracorporeal membrane oxygenation (ECMO). The patient maintained the signs of low output and low flow in ECMO, in addition to poor perfusion in the right lower limb after cannulation. It was therefore decided to replace the ECMO with a ventricular assist system (VAS) (CentriMag; Levitronix LLC, Waltham, MA, United States), on July 1, 2019. The procedure was performed by means of median sternotomy, with decannulation of the ECMO and myocardial biopsy.

During the hospitalization with the device, she received corticotherapy and immunotherapy lasting five days. During this time, she required correction of a pseudoaneurysm in the right femoral artery. In addition, she underwent decompression fasciotomy in a right anterior tibial store because of presentation of compartmental syndrome. She evolved with progressive hemodynamic stability and, on July 8, 2019, she presented aphasia of momentary expression, shown by transcranial doppler microembolization in the left middle cerebral artery. Her anticoagulation was adjusted, but the microembolization was maintained.

**Figure 1.**
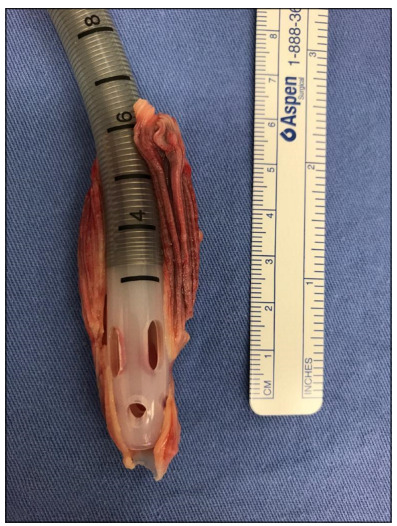
Left ventricular intracavitary thrombus of the device.

On July 11, 2019 (10 days after implantation), she was decannulated from the CentriMag. An intracavitary left ventricle (LV) thrombus from the device (Figure 1) was seen, which evolved with progressive weaning from VAD and corticotherapy, with clear recovery from the bi-ventricular dysfunction (ejection fraction, EF: 17% to 58%) and recovery of strength in the right forefoot. Analysis on the myocardial biopsy confirmed that the cause of the myocarditis was positivity for the Coxsackie virus (viral load of *Erythroparvovirus* and HHV6 type 6B).

**Figure 2.**
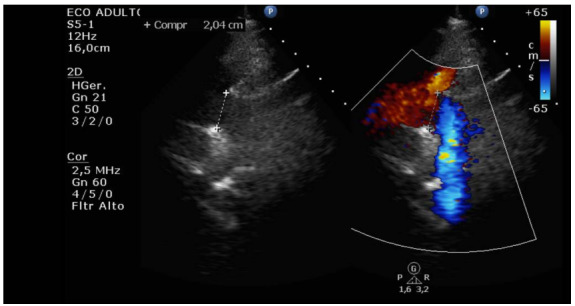
Bi-dimensional echocardiogram with N-flow mapping after explantation of CentriMag device, showing preserved bi-ventricular function (July 16, 2019).

Eight days after implant removal (July 19, 2019), the patient was discharged from the hospital with preserved bi-ventricular function (Figure 2). Currently, the patient is in outpatient follow-up without complaints, presenting good quality of life and preserved heart function (EF 62%).

## DISCUSSION

In this case report, we present a peculiar case of a 35-year-old puerperal woman who progressed with significant worsening of cardiac function within a few hours, even with passage of an IAB and ECMO. The decision to install a CentriMag device in this patient was crucial for enabling myocardial recovery and significant improvement of cardiac output. Reports involving use of CentriMag to treat heart failure in the postpartum period remain rare. Table 1 shows the results from a systematic search for similar studies using the PubMed and EMBASE databases.

**Table 1.** Systematic review of the literature

Database	Search strategy	Results
MEDLINE/PubMed	((“heart failure” [Mesh]) OR “myocarditis”[Mesh]) AND “Heart-assist devices”[Mesh] AND “Postpartum Period”[Mesh]	Case report: 4 Original article: 1
	((heart failure) OR Refractory cardiogenic shock) AND ((centrimag OR heart-assist devices)) AND (postpartum)	Case report: 9 Original article: 15 Review: 3
EMBASE	(‘heart failure’/exp OR ‘heart failure’ OR ‘myocarditis’/exp OR myocarditis) AND (‘centrimag’/exp OR centrimag) AND (‘postpartum’/exp OR postpartum)	Original article: 3 Conference abstract: 4

Temporary mechanical circulatory assist devices (MCADs) are important for re-establishing the hemodynamic condition and should be indicated individually. In addition, they serve as an aid for decision-making up to the point of defining the approach to be taken in cases where immediate hemodynamic support is required (as a bridge to decision); or for recovery of ventricular function in cases of acute myocardial infarction (as a bridge to recovery); or as hemodynamic support and clinical stabilization of patients in a severe condition who are in a transplant queue (as a bridge to transplantation).^4^

It is known that IAB is widely used as the first option for treating heart failure,^5^ but in some cases in which refractory cardiogenic shock occurs, ECMO is an excellent and rapid option. CentriMag provides a temporary option for ventricular function support until the myocardium recovers,^6^ thus preventing low output from leading the heart to irreversible cellular conditions.

Faced with the clinical picture of worsening of our patient’s cardiac function, the decision to implant CentriMag was made in a matter of hours. This was in accordance with the recommendations of the Interagency Registry for Mechanically Assisted Circulatory Support Classification (INTERMACS),^7^ in which use of MCADs is strongly indicated for patients with the profiles INTERMACS 1 (severe cardiogenic shock) and INTERMACS 2 (progressive decline in renal, hepatic, nutritional and lactatemia function, despite use of inotropes).

## CONCLUSIONS

The decision to implant a MCAD should be individualized, as patient profiles do not always match the indications in the guidelines and protocols. In this study, clinical discussion of the case among the medical and multi-professional teams was essential in order to be able to successfully reverse the patient’s severe clinical condition without sequelae, through using a CentriMag implant.
